# First Report of *Aprostocetus asthenogmus* (Hymenoptera: Eulophidae) in South America and Parasitizing Eggs of Triatominae Vectors of Chagas Disease

**DOI:** 10.1155/2014/547439

**Published:** 2014-01-19

**Authors:** Claudiney Biral dos Santos, Marcelo Teixeira Tavares, Gustavo Rocha Leite, Adelson Luiz Ferreira, Leonardo de Souza Rocha, Aloísio Falqueto

**Affiliations:** ^1^Unidade de Medicina Tropical, Universidade Federal do Espírito Santo (UFES), Avenida Marechal Campos 1468, 29043-900 Vitória, ES, Brazil; ^2^Núcleo de Entomologia e Malacologia do Espírito Santo, Secretaria de Estado da Saúde do Espírito Santo, Rua Pedro Zangrande 381, 29164-020 Serra, ES, Brazil; ^3^Laboratório de Biodiversidade de Insetos, Universidade Federal do Espírito Santo (UFES), Avenida Marechal Campos 1468, 29043-900 Vitória, ES, Brazil; ^4^Setor de Parasitologia, Departamento de Patologia, Centro de Ciências da Saúde, Universidade Federal do Espírito Santo (UFES), Avenida Marechal Campos 1468, 29043-900 Vitória, ES, Brazil; ^5^Escola Superior de Ciências da Santa Casa de Misericórdia de Vitória (EMESCAM), Avenida Nossa Senhora da Penha 2190, 29045-402 Vitória, ES, Brazil; ^6^Centro Universitário do Espírito Santo (UNESC), Rua Fioravante Rossi 2930, 29703-855 Colatina, ES, Brazil

## Abstract

We report for the first time the parasitism of eggs of two triatomine Chagas disease vectors, *Triatoma infestans* and *T. vitticeps*, by the microhymenopterous parasitoid *Aprostocetus asthenogmus*. We also describe the first identification of this parasitoid in South America. *A. asthenogmus* were captured near unparasitized triatomine colonies in the municipality of Vitória, state of Espírito Santo, Brazil, and placed into pots with recently laid triatomine eggs. After 24 days, we observed wasps emerging from *T. infestans* and *T. vitticeps* eggs. Several characteristics of this parasitoid species suggest that it could be a potential biological control agent of triatomine species.

## 1. Introduction

Triatominae (Hemiptera, Reduviidae) is a subfamily of hematophagous insects known as “kissing bugs” whose members are principally distributed throughout the Neotropical region [1–3]. Certain triatomine species are responsible for the transmission of *Trypanosoma cruzi* Chagas, 1909 (Kinetoplastida, Trypanosomatidae)—the etiological agent of Chagas disease, which is one of the most dreaded parasitic diseases of Latin America [4].

Attempts to control *T. cruzi* transmission began soon after Carlos Chagas' work in the 1900s. The advent of synthetic insecticides in the 1940s was the first major breakthrough in identifying effective techniques for kissing bug control; the introduction of pyrethroids, a more cost-effective synthetic type of insecticide, was another important advance that occurred in the 1980s [5]. Despite these efforts, high levels of vector-borne transmission still occur in many areas, and several endemic countries had to develop large-scale surveillance and intervention programs [6].

Since the Chagas disease discovery, the biological control of its vectors has been considered [7–10]. However, its application to effective triatomine control in the field is still incipient; little success has been achieved by the use of parasitoid wasps [10], although recent studies with entomopathogenic fungi are promising [11]. Several species clustered in different taxonomic groups might function as triatomine pathogens, predators, or parasitoids [12, 13]. For example, the best-known kissing bug parasitoid *Telenomus fariai* Lima, 1927 (Hymenoptera, Scelionidae), is able to parasitize the eggs of species in three triatomine genera, *Panstrongylus*, *Rhodnius*, and *Triatoma* [12, 14, 15]. Parasitoid wasps are distributed throughout the world in a variety of environments [16] and usually parasitize the eggs of a wide range of insects, many Triatominae species among them [12]. They are considered to have biological, ecological, and economic importance, and some species are employed as pest biological control agents [17, 18].

In this communication, we report for the first time the experimental parasitism of eggs of the triatomine species *Triatoma infestans* (Klug, 1834) and *Triatoma vitticeps* (Stål, 1859) by *Aprostocetus asthenogmus* (Waterston, 1915) (Hymenoptera, Eulophidae). This species is an endophagous egg parasite that has only been reported to parasitize the ootheca of the cockroaches *Periplaneta americana* (Linnaeus, 1758), *Periplaneta australasiae* (Fabricius, 1775), and *Periplaneta brunnea* Burmeister, 1838 (Blattodea, Blattidae) [19, 20]. We also report the first record of *A. asthenogmus* in South America, which has previously been recorded in the Caribbean, India, North Africa, the Palearctic, China, Seychelles, and Sri Lanka [19, 21–24]. We also discuss its potential as a biological control agent of domiciliary triatomine species.

## 2. Materials and Methods

In January 2005, we observed *A. asthenogmus* adult wasps flying near *T. infestans *and *T. vitticeps* colonies established in 2004 at the *Unidade de Medicina Tropical* of *Universidade Federal do Espírito Santo*, municipality of Vitória, state of Espírito Santo, Brazil (20°17′53′′S, 40°18′58′′W). However, the triatomines and their eggs were maintained in mesh-covered pots, preventing any spontaneous wasp-triatomine egg contact. We carried out an investigation to determine if *A. asthenogmus* was able to parasitize triatomine eggs. The adult wasps were captured using a Castro suction device and placed into two pots with recently laid eggs: 11 wasps with 51 eggs of *T. infestans* and 10 wasps with 79 eggs of *T. vitticeps*. The pots were maintained at an environmental temperature (~28°C) and observed daily for wasp emergence.

## 3. Results and Discussion

**Figure 1:**
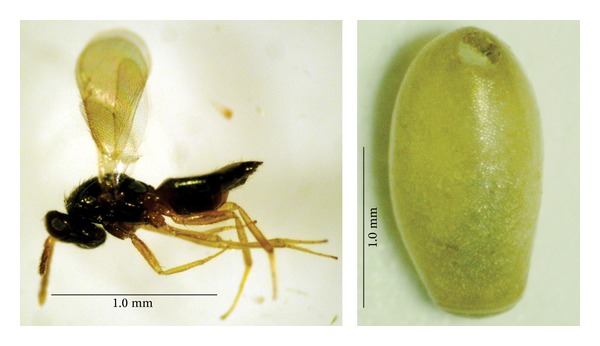
Parasitoid wasp *Aprostocetus asthenogmus* (Waterston, 1915) and *Triatoma vitticeps* (Stål, 1859) egg.

After 24 days, we observed wasps emerging from triatomine eggs. Among *T. infestans *eggs, 29 (56.8%) became parasitized and produced 29 adult wasps, whereas 36 (45.5%) *T. vitticeps* eggs were parasitized and produced 36 adult wasps (Figure 1).

Microhymenoptera of the Aphelinidae, Eupelmidae, Encyrtidae, Pteromalidae, and Scelionidae families were previously reported to parasitize Triatominae eggs naturally and experimentally [7, 12]. However, this is the first report that Eulophidae are able to parasitize triatomine eggs [19, 20].

In cockroaches, *A. asthenogmus* exhibits gregarious habits with a mean of 69.5 parasitoids emerging per ootheca (approximately 9 × 6 mm), which can contain up to 16 eggs, and a mean development time of 43 days [19, 25, 26]. In the two species of triatomine we used, however, *A. asthenogmus* showed a solitary habit, with only one adult emerging from each egg and a shorter development time. These differences could be partially explained by the size variation between triatomine eggs and cockroach oothecas and less resource competition.

*T. vitticeps* has been reported to be parasitized only by *T. fariai*, but *T. infestans* is reportedly parasitized by other microhymenopteran species in addition to *T. fariai* [12, 14, 15]. *T. vitticeps* is a wild and endemic species of the Atlantic Forest that is responsible for sporadic cases of human Chagas disease in that region [27–30], and *T. infestans* is considered the most important domiciliated *T. cruzi* vector that was previously widely distributed in South America [31, 32].

Although it could be considered that the particular laboratory conditions (e.g., scarcity of natural hosts and the confinement imposed) could have stimulated *A. asthenogmus* to use nonnatural hosts, our confirmation of parasitism of triatomine eggs suggests the possibility of this parasitoid acting as a natural enemy of these two species of *T. cruzi* vectors. Several characteristics of this species suggest that it could be a potential biological control agent of triatomine eggs: *A. asthenogmus* was apparently attracted by the triatomine colony, it shows a wide geographical distribution, it is a natural parasitoid of several worldwide cockroach pest species, and it requires no preovipositional period (at least in cockroaches) and accepts 1- to 30-day-old oothecae [25]. It is also promising that the adult wasp developmental time in triatomine eggs was shorter than in cockroach oothecae; moreover, only one adult wasp emerged per triatomine egg, allowing a unique female to parasitize a potentially greater number of triatomine eggs.

## 4. Conclusions

*A. asthenogmus*, first recorded in South America, can parasitize eggs of triatomines, suggesting that it could be a potential biological control agent of these Chagas disease vectors. On the basis of these findings, we suggest that new studies should be carried out to evaluate the host preference plasticity of *A. asthenogmus* in new environments and its potential as a biological control agent of domiciliary and peridomiciliary triatomine species. Experiments evaluating sex ratios, parasitism capacity, dispersal ability, and possible female wasp competition for egg parasitization (including possible egg-marking behavior) should be included in future work.
